# IL-10 Treatment Is Associated with Prohibitin Expression in the Crohn's Disease Intestinal Fibrosis Mouse Model

**DOI:** 10.1155/2013/617145

**Published:** 2013-04-14

**Authors:** C. Yuan, W.-X. Chen, J.-S. Zhu, N.-W. Chen, Y.-M. Lu, Y.-X. Ou, H.-Q. Chen

**Affiliations:** ^1^Department of Gastroenterology, Shanghai Sixth People's Hospital Affiliated to Shanghai Jiao Tong University, Shanghai 200233, China; ^2^Department of General Surgery, Shanghai Sixth People's Hospital Affiliated to Shanghai Jiao Tong University, Shanghai 200233, China

## Abstract

Prohibitin, which can inhibit oxidative stress and mitochondrial dysfunction, has been shown to have significant anti-inflammatory activities. Here, we investigate the effects of altering prohibitin levels in affected tissues in the interleukin-10 knockout (IL-10KO) mouse model with intestinal fibrosis. The aim of this study is to investigate the effects of IL-10 on prohibitin and the role of prohibitin in intestinal fibrosis of murine colitis. After the mice were treated with IL-10, prohibitin expression and localization were evaluated in IL-10KO and wild-type (WT, 129/SvEv) mice. The colon tissue was then investigated and the potential pathogenic molecular mechanisms were further studied. Fluorescence-based quantitative polymerase chain reaction (FQ-PCR) and immunohistochemistry assays revealed a significant upregulation of prohibitin with IL-10 treatment. Furthermore, IL-10 decreases inflammatory cytokines and TGF-**β**1 in the IL-10KO model of Crohn's disease and demonstrates a promising trend in decreasing tissue fibrosis. In conclusion, we hypothesize that IL-10 treatment is associated with increased prohibitin and would decrease inflammation and fibrosis in an animal model of Crohn's disease. Interestingly, prohibitin may be a potential target for intestinal fibrosis associated with inflammatory bowel disease (IBD).

## 1. Introduction

Inflammatory bowel disease (IBD) is a chronic and multifactorial gastrointestinal inflammatory condition that is clinically categorized as ulcerative colitis (UC) or Crohn's disease (CD). IBD fibrosis can occur in both UC and CD, but it is much more prevalent in CD. The etiology and pathophysiology of IBD are still unknown and multifactorial. Typical CD presentations include discontinuous involvement of various portions of the gastrointestinal tract and the development of complications including strictures, abscesses, or fistulas [1, 2]. Current anti-inflammatory therapies neither prevent fibrosis nor reverse established strictures, which may present years after remission of active inflammation.

Prohibitin, which can inhibit oxidative stress and mitochondrial dysfunction, has been shown to have significant anti-inflammatory activities. Prohibitin is a ubiquitously expressed, multifunctional protein implicated in many cellular processes, including mitochondrial function and protein folding [3]. It has been implicated in the regulation of proliferation, cellular apoptosis, and gene transcription [4, 5]. Moreover, prohibitin exhibits a remarkable degree of sequence conservation across species. The protein sequences of mouse and rat prohibitin are virtually identical, and these differ from the human protein sequence by a single amino acid [6]. A recent study showed that prohibitin levels are decreased in Crohn's disease colonic mucosal biopsies [7], and little is known about the regulation and role of prohibitin during intestinal inflammation. Prohibitin has been shown to exhibit an antifibrotic effect in animal models of cirrhosis [8] and renal fibrosis [9]. Current studies with prohibitin focus on the acute phase in inflammation rather than the expression and role in the fibrosis course of IBD.

Of all the cytokines and growth factors involved, transforming growth factor (TGF)-*β*1 is one of the most potent fibrogenic cytokines in not only CD [10] but also in several other fibrotic diseases such as systemic sclerosis and hepatic cirrhosis [11, 12]. Therefore, regulating TGF-*β*1 expression has been investigated as a potential therapeutic for preventing and treating intestinal fibrosis.

*α*-smooth muscle actin (*α*-SMA) is one of six actin family members. In the adult, prominent  *α*-SMA expression can be found in vascular smooth muscle cells and myoepithelial cells. Epithelial-mesenchymal transition (EMT) which contributes to tissue fibrosis is also associated with cells that eventually express  *α*-SMA as myofibroblasts [13].

Interleukin-10 (IL-10) is a pluripotent cytokine that plays a pivotal role in the regulation of immune and inflammatory responses. IL-10 has been shown to suppress the production of proinflammatory mediators and downregulate costimulatory molecules that are critical for the activation of T cells [14]. 

In this study, we used IL-10KO mice that spontaneously form symptoms similar to Crohn's disease as an inflammatory bowel disease model to establish a colon mice intestine fibrosis model. The aim was to detect and investigate if the effects of IL-10 on prohibitin has antifibrotic effects and prevents the progression of intestinal fibrosis in the IL-10KO mice model of CD and to determine the role of prohibitin in intestinal fibrosis of murine colitis.

## 2. Materials and Methods

### 2.1. Materials

We purchased 5-week-old female homozygous IL-10 knockout (IL-10KO) and wild-type (WT) 129/SvEv mice from Jackson Laboratory (SPF). The primers for TGF-*β*1, NF-*κ*B, Nrf2, IL-10, collagen I, and  *α*-SMA were synthesized by ABI Co., Ltd. (USA). All antibodies escape prohibitin were purchased from Santa Cruz Biotechnology, Ltd. (Santa Cruz, CA, USA). Antiprohibitin was obtained from Sigma-Aldrich (St. Louis, MO, USA).

### 2.2. Animals 

Specific pathogen-free (SPF) female homozygous IL-10 knockout (IL-10KO) and wild-type (WT) 129/SvEv mice (Jackson Laboratory) of 5 weeks of age and weighing 19–23 g were used for this study. The animals were randomly allocated into three groups: group A (control group, 18 WT mice), group B (IL-10KO mice group, 18 IL-10KO mice), and group C (IL-10 treatment group, 12 IL-10KO mice). The animals were housed under SPF and temperature-controlled conditions, with light/dark cycles of 12/12 hours and free access to water and standard rodent chow at the animal center of the Sixth People's Hospital affiliated to Shanghai Jiao Tong University.

### 2.3. Ethics

This study was carried out in strict accordance with the recommendations in guidelines for the care and use of laboratory animals. The protocol was approved by the Committee on the Ethics of Animal Experiments of the Shanghai Sixth People's Hospital Affiliated to Shanghai Jiao Tong University (Permit Number: SYXK [Hu] 2011-0128). All surgery was performed under lidocaine anesthesia, and then mouse was sacrificed by cervical dislocation and all efforts were made to minimize suffering.

### 2.4. IL-10KO Colitis Model

Using aseptic techniques, the mice of the treated group and model group were treated with intraperitoneal injections of IL-10 (5 *μ*g/kg body weight, three times per week) and 0.9% physiological saline since week 12. The control group received no treatment; then mice of control group and IL-10KO mice group were sacrificed at weeks 12, 14, and 16, and the treated group was sacrificed at week 14 and 16. Mice were housed until the appropriate age, and then they were sacrificed by cervical dislocation.

In this model, IL-10KO mice developed spontaneous colitis between 6 and 8 weeks. Then, a spontaneous chronic intestinal inflammation and prominent fibrosis occurred at week 12.

### 2.5. Histopathology Staining

Entire colons were removed, fixed in 10% formaldehyde, and embedded in paraffin. Sections were stained with hematoxylin and eosin (H&E) reagent and Masson's trichrome stain.

### 2.6. Collagen Assay

We harvested colonic tissues from IL-10KO and WT mice at 12, 14, and 16 weeks of age. From each tissue sample, one section was obtained by using Masson's original trichrome stain. This trichrome system stains collagen blue, nuclei purple-brown, and cytoplasm pink. Collagen area was defined as the distinct blue color region and was distinguished from muscle, blood, and inflammatory cells. The total length of each tissue section was measured. The collagen area was also measured by using a Sircol Collagen Assay kit according to the manufacturer's instructions. 

### 2.7. Assessment of the Histologic Severity

The histological severity of colitis was evaluated by H&E-stained and coded sections by modifying the validated scoring system described by Tamaki et al. [15]. All slides were scored by a gastrointestinal pathologist in a blinded manner for inflammation based on the scoring system for inflammation (macrophage, lymphocyte, and neutrophil infiltration in the lamina propria or submucosa) was scored for severity as follows: normal, 0; minimal, 1; mild, 2; moderate, 3; marked, 4; and severe, 5. In brief, the inflammation score for a given tissue section is the sum of the scores given to the four regions or features assessed for inflammation.

### 2.8. Real-Time Reverse Transcription Polymerase Chain Reaction

Total RNA was extracted from colon mucosa by using TRIzol reagent (Invitrogen). RT of the RNA was conducted with 200 U of M-MLV RT RNase H-Deletion Mutant (Promega) at 42°C for 60 min by using the 7500 Sequence Detection System (Applied Biosystems). The PCR primer sequences were as follows: Prohibitin, 5′-TGG CGTTAGCGGTTACAGGAC-3′ and 5′-GAGGATGCGTAGTGTGATCTTGAC-3′; Nrf2, 5′-CCTCGCTGGAAAAAGAAGTG-3′ and 5′-GGAGAGGATGCTGCTGAAAG-3′, TGF-*β*l, 5′-TGAGCACTGA AGCGAAAGCC-3′ and 5′-GATTCAAGTCAACTGTGGAGCAAC-3′, collagen I, 5′-AATGGTGCTCCTGGTATTGC-3′ and 5′-CTCCTTTGGCACCAGTGTCT-3′,  *α*-SMA, 5′-TGCTGTCCCTCTATGCCTCT-3′ and 5′-GAAGGAATAGCCACGCTCAG-3′ and GAPDH, 5′-GGTATCCTGACCCTGAAGTA-3′ and 5′-GGGGTGTTGAAGGTCTCAAA-3′. For PCR, after an initial incubation for 30 s at 95°C, 40 cycles were performed consisting of 5 s at 95°C, 5 s at 60°C, and 30 s at 72°C for extension. Relative differences in mRNA expression were determined using the 2^−ΔΔCT^ method. Briefly, the cycle number at which the transcript being analyzed became detectable (CT) was normalized to the cycle number at which the GAPDH gene transcript was detected, referred to as  ΔCT.

### 2.9.  Immunohistochemistry Staining

For immunohistochemical analysis of prohibitin and  *α*-SMA colon tissue fixed with 4% buffered paraformaldehyde was embedded in paraffin, and 4 *μ*m-thick sections were stained. After deparaffinization, antigen retrieval was performed by immersing the section in 10 mM citrate buffer (Ph 6.0) and heating twice in a microwave oven (95°C) for 5 min each time. Endogenous peroxidase activity was blocked by incubation with 1% hydrogen peroxide in distilled water for 10 min. All sections were then incubated with antiprohibitin and anti-*α*-SMA antibody. After incubation with second antibody immunoglobulin, the sections were stained with diaminobenzidine. The sections were counterstained with hematoxylin. Then, we used the image analysis software to measure the integral optical density (IOD) of prohibitin and  *α*-SMA. 

### 2.10. Statistical Analysis

Data analysis was performed using SPSS version 18.0 (SPSS, Chicago, IL, USA) statistical software. The results are shown as mean ± standard deviation. One-way analysis of variance (ANOVA) was used to analyze the differences between groups. Nonparametric data were analyzed by Kruskal-Wallis and Mann-Whitney  *U*  tests and Pearson's correlation coefficient was used to determine the relationships between the indicators. *P* < 0.05 was considered significant. 

## 3. Results

### 3.1. Effects of IL-10 on Animal Weight

**Figure 1:**
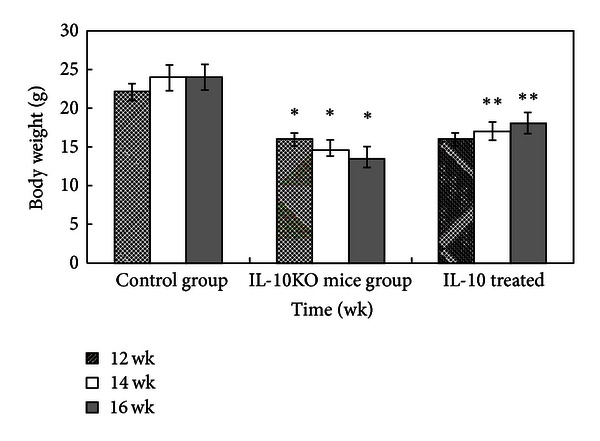
Effects of IL-10 on animal weight level in mice. Data were expressed as mean of weight values ± SD of 6 mice in each group. *P < 0.05 compared with control group; **P < 0.05 compared to the IL-10KO mice group.

There were significant differences in the mean weight of the control group, IL-10KO mice group, and IL-10-treated group in all of the experiments. The IL-10KO group exhibited a marked decrease in their body weight as compared to the control group at 12, 14, and 16 weeks (*P* < 0.05). The mice were sacrificed on wk 12, 14, and 16 and various degrees of edema and adhesion were found over the distal colon in a length of 3–5 cm and 1–3 cm in the model group and IL-10KO treatment group, respectively. Severe strictures associated with the dilatation of the proximal segment were exhibited gradually over time. In contrast, mice in the control group had only minimal inflammatory change. Administration of IL-10 led to a significant increase in body weight at 14 and 16 weeks as compared to the IL-10KO mice group (*P* < 0.05). At the end of study, the surviving mice in the IL-10-treated group gradually regained their weight but still failed to reach the initial weight (Figure 1). 

### 3.2. Histopathological Abnormalities in the Colon 

**Figure 2:**
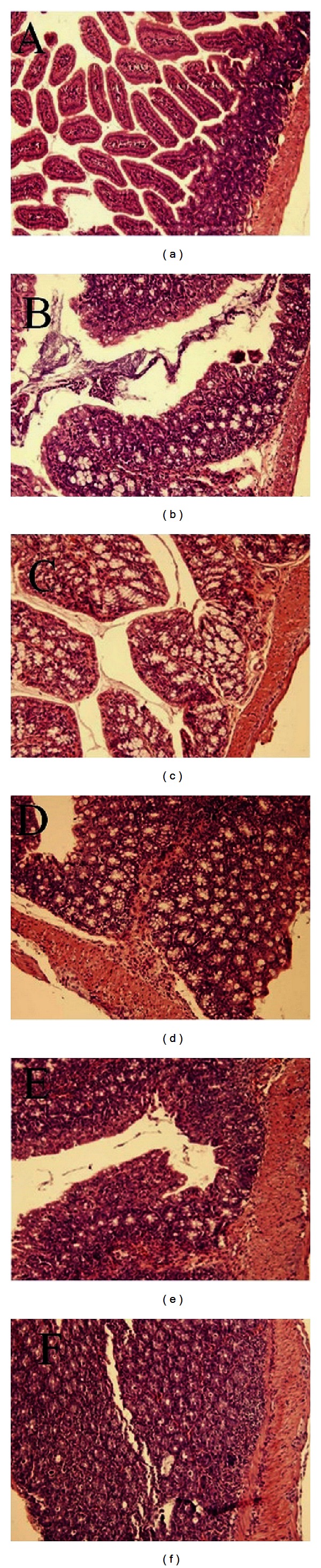
Histological findings of IL-10KO and WT mice by H&E staining (×200). Colonic sections of WT mice (a), IL-10 treatment mice at 14 (b) and 16 (c) wk of age and IL-10KO mice at 12 (d), 14 (e), and 16 (f) wk were stained with H&E staining.

**Figure 3:**
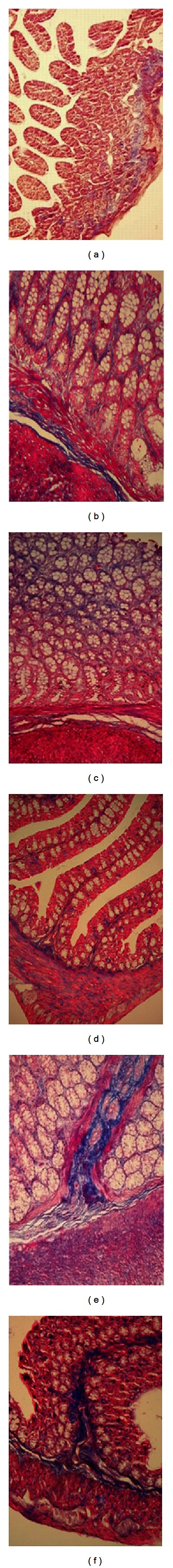
Masson's trichrome stain (×400) indicates that collagen deposition (blue) is located mainly in the mesenchymal, mucosa layer, submucosal areas, and muscularis propria in IL-10KO mice at 12 (d), 14 (e), and 16 (f) wk. Little collagen deposition was found in the mesenchymal layer in WT mice (a). IL-10KO treated with IL-10 5 *μ*g/kg body weight, three times per week at 14 (b) and 16 (c), have less collagen in the submucosal areas and muscularis propria.

**Figure 4:**
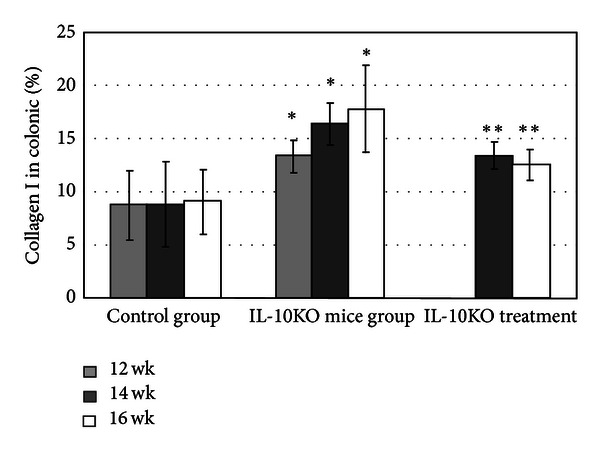
Trichrome image analysis: collagen I in colonic (%) *P < 0.05, versus control group; **P < 0.05, versus IL-10KO mice group.

The H&E staining histologic findings revealed colonic epithelial hyperplasia, crypt abscess, glands arranged in disorder, and the forming of edema and ulceration of submucosa in the propria of the colonic tissue from IL-10KO mice at 12, 14, and 16 weeks of age (Figures 2(d)–2(f)). IL-10 treatment led to a significant amelioration of colonic epithelial hyperplasia and cellular infiltration with IL-10 treatment (Figures 2(b) and 2(c)). The histopathological colitis score in the IL-10KO mice group was significantly increased with age and was greater in IL-10KO mice than in the control group at each time point. In treatment group, the histopathological colitis score was decreased significantly than those in IL-10KO mice group at week 16 (Table 1, *P* < 0.05). Masson's trichrome stain sections of the colon were analyzed for collagen content as described in Section 2 (Figure 4). In WT mice, little collagen deposition was found in the mesenchymal layer. On the other hand, collagen deposition in IL-10KO mice was localized not only in the mesenchymal but also in the mucosa, submucosal, and muscularis propria areas. We then found that collagen deposition was increased with age, specifically at 12-, 14-, and 16-week time points (Figures 3(d)–3(f)). Furthermore, the amount of collagen deposited in the submucosal areas and muscularis propria was markedly reduced in the IL-10-treated group compared to that in the IL-10KO mice (Figures 3(b) and 3(c),  *P* < 0.05). 

### 3.3. Prohibitin, Nrf2, Collagen I,  *α*-SMA, and TGF-*β*1 Gene Expression in Colonic Tissues of Wild-Type and IL-10KO Mice 

The gene expressions of TGF-*β*1, collagen I, and  *α*-SMA in the IL-10KO mice group were upregulated in a time-dependent manner and were much more significant compared to those in the control group. In addition, gene prohibitin expression of Nrf2 in the colonic tissue of IL-10KO mice was down-regulated in a time-dependent manner. Moreover, prohibitin and Nrf2 mRNA expression were significantly up-regulated and TGF-*β*1,  *α*-SMA mRNA expression was markedly reduced in the IL-10-treated group when compared with the IL-10KO mice group at 14, 16 wk (Table 2).

### 3.4. Effects of IL-10 on Protein Expression of Prohibitin and  *α*-SMA

**Figure 5:**
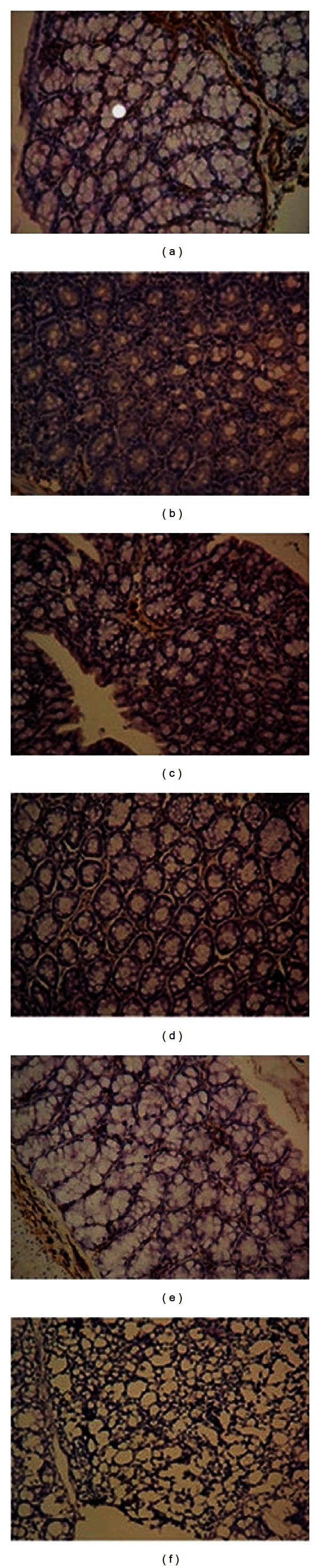
Effects of IL-10 on protein expression of prohibitin (×250). Colonic sections of WT mice (a), IL-10 treatment mice at 14 (b) and 16 (c) wk of age and IL-10KO mice at 12 (d), 14 (e), and 16 (f) week were stained with.

We further detected protein expression levels of prohibitin and  *α*-SMA in colonic tissues by measuring the integral optical density. The protein levels of prohibitin were lower in the IL-10KO mice group than in the control group (Figures 5(d)–5(f)) and that of  *α*-SMA in the IL-10KO mice group was much more significant compared with that in the control group. Moreover, there was a significant difference in prohibitin and  *α*-SMA in the treatment group and the IL-10KO mice group. Prohibitin protein expression levels were significantly up-regulated (Figures 5(b) and 5(c)) and  *α*-SMA was markedly reduced in the IL-10-treated group when compared with the control group at 14 and 16 weeks (Table 3). Protein expression of prohibitin was negatively correlated with the histologic colitis score (*r* = −0.859, *P* < 0.01) and  *α*-SMA protein expression (*r* = −0.798, *P* < 0.05).

## 4. Discussion

In IBD, especially in the CD, long-term recurrent intestinal chronic inflammation and excessive damage repair could cause intestinal tract fibrosis and intestinal stricture. Research shows that about a third of the CD patients developed stenosis of bowel and needed surgery. However, after the surgery, about 70% of the postoperative patients developed stenosis of the bowel [16]. Immunomodulators and biologic therapies are the therapeutic mainstay for CD. These therapies are highly effective in treating inflammation in most patients. However, their effects have not specifically shown to decrease fibrosis. The findings in the present study demonstrated that prohibitin is critically involved in the development of organ fibrosis and indicated that regulation of prohibitin might prevent and alleviate intestinal fibrosis associated with human IBD.

The Masson collagen trichrome staining in IL-10KO mice showed massive amounts of collagen deposition, mainly in the lamina propria, submucosal areas, and muscularis propria in the colonic tissues of IL-10KO mice with colonic inflammation, and the amounts increased with age. In addition, we found that the gene expressions of TGF-*β*1,  *α*-SMA, and collagen I in IL-10KO mice were markedly increased when compared to those in WT mice, especially at 16 wk. However, the expression of prohibitin and Nrf2 in IL-10KO mice was markedly reduced over that of WT mice. Protein expression of prohibitin negatively correlated with the histologic colitis score and  *α*-SMA protein expression. Decreased prohibitin levels was associated with increased ECM (extracellular matrix) levels (*α*-SMA and collagen I). Theiss et al. [17] found that the therapeutic delivery of prohibitin to the colon reduces the severity of DSS-induced colitis in mice. These findings suggested that chronic intestinal inflammation of IL-10KO mice reduced prohibitin, resulting in intestinal fibrosis. 

In this investigation, we found that IL-10 treatment in mice was associated with increased expression of prohibitin and Nrf2, attenuation of the lesion in the intestinal fibrosis, and significant reduction in the expressions of TGF-*β*l,  *α*-SMA, and collagen. We also found that in IL-10-treated mice at 16 wk in histological and immunological findings were significantly improved, and colonic mucosa edema was lowered and the degree of disorder in the intestinal mucosa gland arrangement was reduced. These results show that IL-10 can ameliorate intestinal fibrosis; this effect is probably related to the regulation of prohibitin by IL-10.

The human prohibitin gene was first found by Sato et al. [18]. Prohibitin is a highly conserved, ubiquitously expressed, multifunctional protein whose expression is decreased during IBD [19]. Additionally, prohibitin is regarded as an apoptosis-regulating protein [20]. The best characterized function of the prohibitin is as a chaperone involved in the stabilization of mitochondrial proteins. It is also thought to play a role in maintaining normal mitochondrial function and morphology. Berger and Yaffe [21] found that loss of function of prohibitin leads to altered mitochondrial morphology, loss of normal reticular morphology, and disorganized mitochondrial distribution. Theiss et al. [17] reported that the elevation of prohibitin in the surface epithelial cells of the colon could reduce the severity of colitis in mice, suggesting that prohibitin may be a novel therapeutic target for inflammatory bowel disease. The results from the abovementioned studies suggest that prohibitin is associated with cell/tissue injury. Similarly, these data support our results. Correlation analysis showed that prohibitin protein expression was negatively correlated with the histologic colitis score and protein expression of  *α*-SMA. Therefore, prohibitin regulation may prove to be a major therapeutic target for treating intestinal fibrosis in human IBD. 

Of all the cytokines and growth factors involved, TGF-*β*1 is known as one of the most potent fibrogenic cytokines in several fibrotic diseases [11–13, 22]. TGF-*β*1 plays a pivotal role in the processes of intestinal fibrosis. Therefore, we examined TGF-*β*1 expression in the colonic tissue of IL-10KO mice. As expected, TGF-*β*1 expression was upregulated in a time-dependent manner in the colonic mucosa. The present study has shown the importance of regulating the local production of TGF-*β*1 as a therapeutic strategy for preventing and suppressing intestinal fibrosis [23]. However, TGF-*β*1 also has diverse important biologic functions such as immunosuppressive function, enhancement of tissue regeneration, and wound healing [24, 25]. In the progression of the fibrosis processes, it cannot only increase the secretion of extracellular matrix, but it can also prevent fiber activator generation. Therefore, TGF-*β*1 may not be an ideal target for preventing fibrosis in patients with inflammatory bowel disease.

We therefore investigated another transcription factor in chronic intestinal inflammation, Nrf2, a transcriptional regulator of antioxidant responses. Nrf2 also plays a pivotal role in the endogenous defense against oxidative stress [26]. Nrf2 is very sensitive to the cellular oxidation reduction status; therefore, any changes in the cellular oxidation reduction can lead to the change of the Nrf2 transcriptional regulation action [27]. Lastres-Becker et al. [28, 29] found that transcriptional activity of Nrf2 increases with low or moderate doses of TNF-*α*  and decreases with high doses of TNF-*α*. Another study [17] showed that prohibitin is the regulator of Nrf2 and can sustain activation of the Nrf2, which can decrease oxidative stress and colitis. In this investigation, we found similar results that prohibitin acts as a regulator of antioxidant response and regulation of prohibitin can sustain activation of the Nrf2. Therefore, these findings indicate that regulation of prohibitin might prevent the development of therapeutic agents for intestinal fibrosis in human IBD.

IL-10 is a cytokine with anti-inflammation and anti-fibrosis characteristics. García-Prieto et al. [30] found that inhibiting the Matrix metalloproteinase-8 (Matrix metalloproteinases-8, MMP-8) can enhance IL-10 expression and increase bleomycin-induced pulmonary fibrosis in rat models. In this experiment, we observe the effect of IL-10 on the intestinal fibrosis in IL-10-treated mice. Theiss et al. [17] reported that improving prohibitin expression in intestinal epithelial cells can effectively relieve the degree of colonic inflammatory reaction in mice. In this study, our results were similar to those in previous studies that showed that IL-10 exhibits an inhibitory effect on the intestinal fibrosis process. Moreover, there is a strong correlation between IL-10 administration and prohibition expression, which ultimately slows fibrosis formation.

Our data revealed that administration of IL-10 treatment remarkably decreased collagen deposition in the colonic tissues of IL-10KO mice. Therefore, we demonstrated that regulation of prohibitin plays an important role in preventing and ameliorating intestinal fibrosis related to intestinal inflammation.

In conclusion, lowered expression of prohibitin is associated with intestinal fibrosis progression, and IL-10 treatment is associated with increased prohibitin in IL-10KO mice. Based on these findings, regulation of prohibitin may be a promising option for the treatment of intestinal fibrosis related to IBD in the future. However, cell culture and further investigations should be conducted to investigate the detailed mechanism.

## Figures and Tables

**Table 1:** The histologic colitis score in each group mice ($\overset{-}{x}\pm s$).

Group-week	*n*	Histologic colitis score
12 wk		
Control	6	1.33 ± 1.21
IL-10KO mice	6	5.67 ± 0.82^a^
14 wk		
Control	6	1.17 ± 0.75
IL-10KO mice	6	6.33 ± 0.82^a^
IL-10 treated	6	5.17 ± 0.75^a^
16 wk		
Control	6	1.50 ± 1.38
IL-10KO mice	6	7.00 ± 0.90^a^
IL-10 treated	6	4.33 ± 0.82^ab^

^a^ *P* < 0.05, versus control group; ^b^ *P* < 0.05, versus IL-10KO mice group.

**Table 2:** Gene expression of prohibitin, Nrf2, collagen I, *α*-SMA, and TGF-*β*1 $\left(\overset{-}{x}\pm s,\%\right)$.

Group-week	*n *	Prohibitin	TGF-*β*1	Nrf2	Collagen I	*α*-SMA
12 wk						
Control	6	4.60 ± 0.72	0.32 ± 0.15	1.58 ± 1.01	0.45 ± 0.24	0.36 ± 0.43
IL-10KO	6	0.74 ± 0.15^a^	1.12 ± 0.22^a^	0.92 ± 0.42^a^	1.13 ± 0.21^a^	0.98 ± 0.48^a^
14 wk						
Control	6	5.10 ± 1.32	0.28 ± 0.16	1.21 ± 0.64	0.40 ± 0.33	0.47 ± 0.13
IL-10KO	6	0.53 ± 0.22^a^	1.48 ± 0.24^a^	0.46 ± 0.27^a^	1.68 ± 0.51^a^	1.58 ± 0.36^a^
IL-10 treated	6	1.85 ± 0.41^ab^	0.62 ± 0.12^ab^	1.09 ± 0.49^ab^	1.10 ± 0.49^ab^	1.18 ± 0.11^ab^
16 wk						
Control	6	4.94 ± 1.05	0.31 ± 0.20	1.62 ± 0.85	0.42 ± 0.24	0.42 ± 0.33
IL-10KO	6	0.42 ± 0.03^a^	1.91 ± 0.55^a^	0.32 ± 0.15^a^	1.88 ± 0.27^a^	1.91 ± 0.95^a^
IL-10 treated	6	2.51 ± 0.28^b^	0.33 ± 0.10^b^	2.13 ± 1.04^b^	0.76 ± 0.25^ab^	0.81 ± 0.50^ab^

^a^ *P* < 0.05, versus control group; ^b^ *P* < 0.05, versus IL-10KO mice group.

**Table 3:** Protein expression of prohibitin and *α*-SMA ($\overset{-}{x}\pm s$).

Time (wk)	Prohibitin			*α*-SMA		
	Control	IL-10KO mice	IL-10 treatment	Control	IL-10KO mice	IL-10 treatment
12	7792.83 ± 666.11	2402.17 ± 936.33^a^		13328.83 ± 5075.65	23903.33 ± 5645.83^a^	
14	7913.57 ± 474.08	1680.33 ± 367.01^a^	3061.33 ± 473.84^b^	15570.00 ± 5249.14	42154.50 ± 8708.8^a^	27734.50 ± 5305.67^b^
16	7618.77 ± 523.92	1259.83 ± 154.16^a^	5491.83 ± 953.40^b^	17103.50 ± 6522.18	50467.33 ± 5074.69^a^	23245.00 ± 4178.07^b^

^a^ *P* < 0.05, versus control group; ^b^ *P* < 0.05, versus IL-10KO mice group.
